# A novel approach towards development of real time chemical dosimetry using pulsating sensor-based instrumentation

**DOI:** 10.1007/s10967-013-2531-x

**Published:** 2013-05-14

**Authors:** N. Malathi, P. Sahoo, K. Praveen, N. Murali

**Affiliations:** Innovative Instrumentation Section, Real Time Systems Division, Electronics Instrumentation and Radiological Safety Group, Indira Gandhi Centre for Atomic Research, Kalpakkam, 603102 Tamilnadu India

**Keywords:** Real time chemical dosimeter, Pulsating conductivity meter, *G* value of HCl, Fricke dosimetry, Absorbed radiation dose

## Abstract

The paper presents an innovative approach towards development of real time dosimetry using a chemical dosimeter for measurement of absorbed radiation dose in the range between 1 and 400 Gy. Saturated chloroform solution in water, a well known chemical dosimeter, is used to demonstrate the concept of online measurement of radiation dose. The measurement approach involves online monitoring of increase in conductivity of saturated chloroform solution due to progressive build up of traces of highly conducting HCl during exposure to gamma irradiation. A high performance pulsating sensor-based conductivity monitoring instrument has been used to monitor such real time change in conductivity of solution. A relation between conductivity shift and radiation dose has been established using radiochemical yield value (*G* value) of HCl. The *G* value of HCl in saturated chloroform dosimeter has been determined using laboratory developed pulsating sensor-based devices. In this connection dose rate of Co-60 gamma chamber was determined using Fricke dosimeter following a simple potentiometric measurement approach developed in-house besides conventional spectrophotometry. Results obtained from both measurement approaches agreed well. Complete instrumentation package has also been developed to measure real time radiation dose. The proposed real time radiation dosimeter is successfully tested in several measurement campaigns in order to assure its performance prior to its deployment in field.

## Introduction

A variety of techniques used for measurement of radiation dose are broadly divided into two categories, such as primary and secondary measurement techniques. The former includes ionization chamber (IC) and calorimeter techniques whereas the latter includes solid state detectors—thermoluminescence detector (TLD), optically stimulated luminescence (OSL), radiochromic films, alanine ESR dosimeter, chemical dosimeters and biological dosimeters, etc. IC [1, 2] is widely used as dosimeter for precise measurement of radiation dose. Such chambers are constructed in various designs for different applications including radiotherapy. The operation of IC is based on ionization of a gas between two electrodes. Though this technique is considered as highly reliable technique with high precision and better accuracy it has certain limitations. ICs are of relatively large physical size and they need high bias voltage for acceptable collection of charges. Unlike chemical dosimeters they are used to measure exposure dose and a conversion factor is used to express it in absorbed dose. Cavity ICs are very compact type and they are used for high energy use. These can be used for real time measurement of radiation dose by connecting the chamber to an electrometer. GM counters are also used in radiation survey meters to measure X- and gamma ray fields in radiation protection application [1]. They offer several advantages such as (i) they are inexpensive, versatile in their construction and geometry, (ii) they practically do not require any amplification or very small amplification if needed. They exhibit strong photoelectric effect response below ~100 keV which restricts their use for high dose measurement.

The measurement of the temperature rise in calorimetric dosimeters provides a direct method for measurement of absorbed dose. National Institute of Standards and Technology, USA recommended two types of calorimeters, water calorimetry and graphite calorimetry measurements, as calibration standards in national level [3]. In spite of this, the use of calorimeter for routine applications suffers a major drawback. Thermal insulation and instrumentation for thermal control as well as measurement obstruct the apparatus to be a portable one for field application. In addition, for low dose rate measurement thermal leakage in and out of the calorimeter sensitive volume limits the accuracy and precision in measurement. This technique is mostly used as a technique to check on other [1, 4]. The use of thermoluminescence as a method for dosimetry for ionizing radiation is an established technique and it is successfully used for last several decades [5]. A TLD has some advantages such as low dependence response to photon energy, linear response for a wide measurement range besides being small size and low cost. It has some disadvantages such as sensitivity to environmental conditions, fading due to temperature and light effect and unsuitability for real time measurements. Radiographic and radiochromic films [3, 6] are also used as dosimeters for direct measurement of absorbed dose. Radiographic films are highly sensitive to light and require a chemical processing and their response depends on radiation energy. Such problems are overcome with the evolution of radiochromic dosimeters. Some of the disadvantages of such dosimeter are (i) nonlinear response with dose and dependent on scanner orientation, (ii) sensitive to temperature, relative humidity, ultraviolet (UV) light, and (iii) unsuitability for online measurement. OSL radiation dosimeter is considered as a very sensitive technique for dose measurement from 0.1 to 100 Gy. The OSL film in combination with optical fiber constitutes a dosimeter probe of relatively small size and it needs small power consumption. It is immune to radiofrequency and electrical interference. Investigations on the suitability of such dosimeter for real time measurement have been pursued by Yan-Ping et al. [7]. Alanine EPR dosimetry has been applied successfully for measurement of intermediate and high radiation doses. Though it is robust and has a high radical stability its sensitivity is very low. In order to improve the sensitivity of EPR dosimeter Gustafsson et al. [8] introduced deuterated ammonium formate as an EPR dosimeter material in place of alanine. This dosimeter is not yet reported for real time measurement of radiation dose. Silicon-based semiconductor and metal oxide field effect transistor dosimeters have been used for quantitative measurement of absorbed radiation dose and in the quality assurance program [9, 10]. Such dosimeters have the advantage of small size and they can be used for in vivo applications. They need several corrections factors for use in field measurement.

Chemical dosimeters, although considered as secondary dosimeters, are used extensively for monitoring absorbed radiation dose. Some of the reported chemical dosimeters used in various ranges are Fricke dosimeter, cerric–cerrous, organic dosimeters such as saturated chloroform solution in water, dual phase chloroform water system (50 % chloroform and 50 % distilled water), organic acids such as oxalic acid, ethanol chlorobenzene, etc. [3, 4]. A new biochemical radiation dosimeter using bilirubin chloroform solution has been reported for reliable measurement of radiation dose in 0–100 Gy range [11]. The most reliable chemical dosimeter is Fricke dosimeter which is widely used for calibration of gamma irradiation chamber [1, 4, 12]. Many researchers conducted elaborate investigations to find different aspects such as source of error in the application of Fricke dosimetry [13], the temperature dependence of *G*(Fe^3+^) [14], effect of sulfuric acid concentration in solution containing oxygen [15], marginal difference in *G*(Fe^3+^) value for Co-60 gamma rays and that for high energy X-rays [16], etc. Olszanski et al. [17] reported various precautionary steps in preparation of reagents, handling of chemicals, instrumentation, development of software to control the read out of Fricke sample and they were successful in bringing uncertainty in measurement between 0.05 and 0.15 %. Hence the Ionizing Radiation Standards Fricke dosimetry system is successfully used as a calibration and measurement technique in Institute for National Measurement Standards, National Research Council, Ottawa. In the present work we used this Fricke dosimetry to evaluate our proposed online radiation dosimetry.

All these chemical dosimeters are successfully used for offline measurement of radiation dose. Since the *G* values (radiochemical yield value) in many of the chemical dosimeters are reported, one can use them for reliable measurement of radiation dose. In order to use these chemical dosimeters for real time measurement of absorbed dose the following difficulties are encountered (i) chemical steps involved in pretreatment of irradiated solution for concentration measurement, and (ii) involved instrumentation for quantitative measurement of small radiation induced physico-chemical changes. Hence chemical dosimeters are not yet promoted for real time application. Moreover the brief review of measurement techniques mentioned above for radiation dosimetry [1–17] shows that there is lack of simple, cost effective but high precise and sensitive technique for real time measurement of absorbed radiation dose. These difficulties were overcome by deployment of a chemical dosimeter with complete instrumentation package for real time measurement of absorbed radiation dose in much simpler way. In order to do so we selected a chemical dosimeter (saturated chloroform solution in water) and adopted a simple online measurement approach to capture small shift in conductivity of such solution during irradiation using our in-house made pulsating conductivity meter [18–20]. Small shift in conductivity in irradiated saturated chloroform solution is due to formation of HCl which is highly conducting. This is explained by following reactions [4].

$$\begin{array}{l} {\text{CHCl}}_{3} + {\text{H}} \to {\text{H}}^{ + } + {\text{Cl}}^{ - } + {}^{\bullet }{\text{CHCl}}_{2}, {\text{CHCl}}_{3} + {\text{OH}} \to {}^{ \bullet }{\text{CCl}}_{3} + {\text{H}}_{2} {\text{O}}, {}^{ \bullet }{\text{CCl}}_{3} + 2{\text{H}}_{2} {\text{O}} \to 3{\text{HCl}} + {\text{COOH}}. \\ \end{array} $$

The first two reactions are induced by gamma irradiation. The free radical (^•^CCl_3_) which is formed in second reaction reacts with water to form highly conducting specimen, HCl. Hence in the present work real time monitoring of conductivity shift during gamma irradiation is explored to develop a simple technique for online measurement of radiation dose. The paper presents the design of dosimeter system with the associated instrumentation for dose measurement, performance of the online dosimeter, determination of *G*(HCl) in saturated chloroform system and an electrochemical measurement approach for Fricke dosimetry.

## Basic principle of measurement

Traces of HCl (a few μM concentration levels) produced in saturated chloroform solution causes minor increase in conductivity which can be measured by deployment of a high performance (high resolution, high precision and fast response) conductivity meter. This is realized by deployment of a high resolution conductivity meter with pulsating sensor-based instrumentation. The increase in conductivity is directly related to the absorbed dose. Hence online measurement of radiation dose is possible by deployment of such pulsating conductivity meter. In the present work in order to measure the change in conductivity a dedicated standalone embedded system with LCD (liquid crystal display) and control-output is designed and fabricated for online radiation measurement. A compatible firmware is designed to measure the change in conductivity in the chemical dosimeter exposed to gamma rays and convert it to absorbed dose.

## Sensing approach

Reliable measurement of radiation dose strictly depends upon the precise and accurate measurement of minor increase in conductivity due to build up of traces HCl during irradiation. Hence for measurement of conductivity at such a low level we adopted a novel approach of conductivity measurement using an in-house built pulsating sensor-based conductivity meter. The principle of measurement technique is described in our earlier publications [18–20]. In this case a mini conductance cell, with a pair of platinum electrodes as sensing electrodes, containing saturated chloroform is placed in a specially designed logic gate oscillator circuit (LGO) which is powered by 5 V DC. With change in conductance of solution during irradiation the digital pulse frequency which is the output of LGO changes. Hence pulse frequency is directly related to shift in conductivity which in turn gives information of radiation dose. Unlike conventional sensors, these sensors generate the first electronic response in digital domain, avoiding intermediate signal processing stages of pre-amplification, post-amplification, analog to digital conversion, etc. Since the primary signal is in digital domain it helps to get excellent noise immunity. This advantage of measurement technique helps to get highly stable output without losing the loss of signal with transmission to a long distance (about 200 m). Thus it is suitable for online field application.

The relation between frequency (*f*) and resistance (*R*) in this timing oscillator circuit is given by the following equation.

1 $$ f\, \propto \,1/RC. $$

For conductance-based sensors, capacitance (*C*) is kept constant and *R* is variable. Since *R* = 1/*K*, where *K* is the conductance (1) can be presented as

2 $$ f\, \propto \,K. $$

Since,

3 $$ K = \kappa /x, $$

where *κ* is the conductivity and *x* is the cell constant (2) can be presented as

4 $$ f\, \propto \,\kappa . $$

The relation between pulse frequency and conductivity is pre-determined following a multipoint calibration technique using a series of KCl standards. In the present work with increase in radiation dose, conductivity increases. Hence, shift in conductivity with respect to blank value (saturated chloroform before irradiation) is a measure of absorbed radiation dose which can be computed using *G* value of HCl (refer “Results and discussion” section).

## Experimental

### Chemicals

Chloroform (ExcelaR grade, purity 99.7 %) was used to prepare saturated chloroform solution in water. Water used in this work was Millipore water, conductivity less than 1 μS cm^−1^. All other reagents such as ferrous ammonium sulphate, ferric ammonium sulphate, sodium chloride, disodium salt of EDTA and potassium dichromate were of AR grade above 99.5 % purity. Sulphuric acid of GR grade (98 % purity) was used. Both ferrous and ferric solutions were standardized against standard potassium dichromate and disodium salt of EDTA solutions, respectively using potentiometric titration.

### Design and fabrication of experimental set up

A miniaturized Perspex cell was used as dosimeter cell which contained about 2.5 mL of saturated chloroform in water. A pair of sensing electrodes (1 mm dia stainless steel [SS] rod, distance of separation between two electrodes: 3 mm, length of exposed portion: 5 mm) was vertically placed into the cell. The cell was externally placed in the timing circuit of LGO. During irradiation the cell was placed inside a Co-60 gamma chamber.

### Instrumentation

#### LGO circuit

**Fig. 1:**
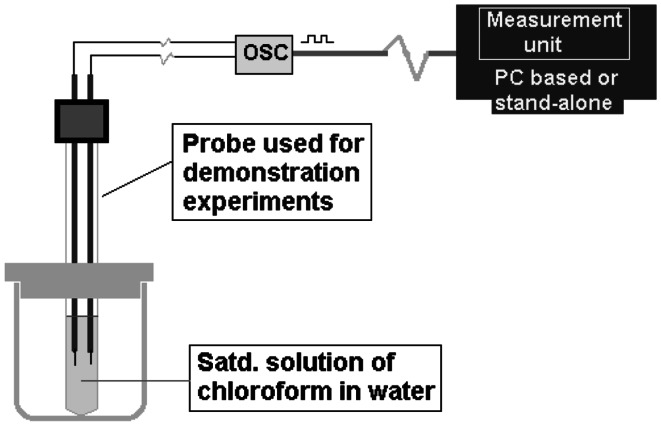
Block diagram of measurement set up

**Fig. 2:**
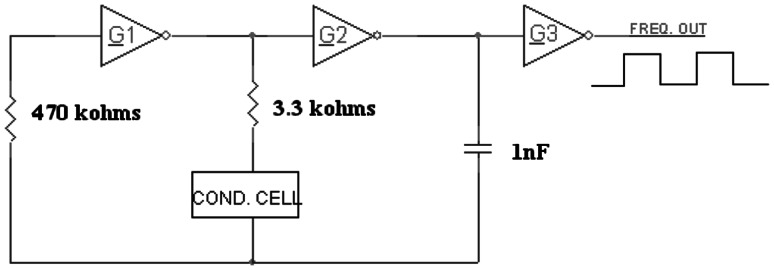
Schematic diagram of oscillator circuit

The sensing electrodes which are a pair of SS rods are serially connected to a fixed resistor (3.3 kΩ). The entire resistance assembly (sensing electrodes which offer variable resistance with change in conductance of solution and the fixed resistance) constitutes the resistance component (*R*) of the timing circuit of an RC oscillator. A fixed capacitor (1 nF) is placed in the LGO which is the capacitive component (*C*). The LGO is powered by 5 V DC. Depending upon the change in conductivity due to formation of HCl, the resistance offered by the liquid varies which in turn is the measure of conductivity of the liquid. The shift in conductivity from the blank solution (pre-irradiated solution) is a measure of radiation dose. The connectivity of conductance probe with the oscillator and the schematic diagram of oscillator circuit are given in Figs. 1 and 2, respectively.

#### Data acquisition cum control system

**Fig. 3:**
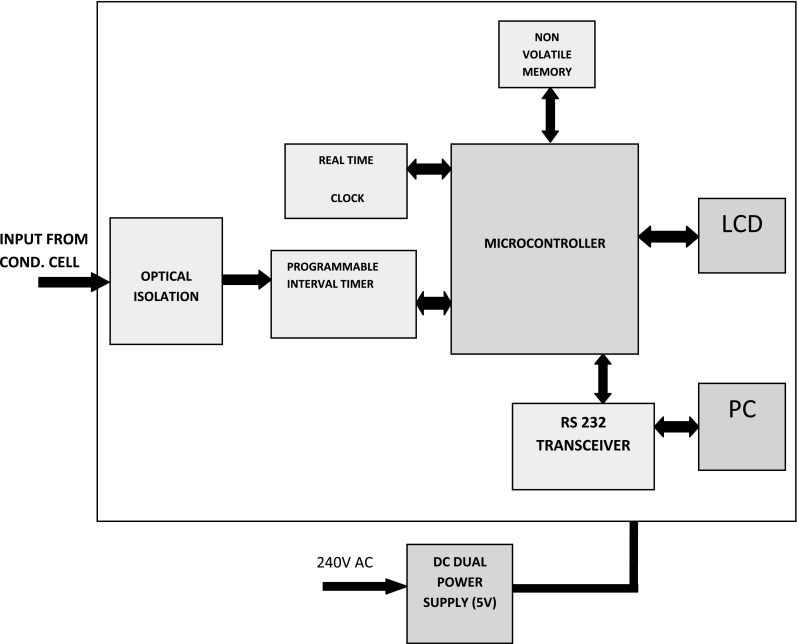
Block diagram of data acquisition device

A standalone data acquisition cum control system is specially designed for measuring the absorbed radiation dose using a chemical dosimeter. The design constitutes hard- and firmware. The hardware consists of optical isolator, programmable interval timer (PIT), microcontroller, real time clock (RTC), memory chip, serial port interface and LCD. Figure 3 shows the block diagram of the data acquisition device. The digital pulse from the conductivity cell is fed to PIT after optical isolation and signal shaping. The timer receives the isolated digital pulse frequency from the LGO in response to the change in conductivity of the physico-chemical medium. An 8051-based CMOS controller AT89C55WD is the brain of the system which is interfaced with PIT, LCD, RTC, EEPROM and Serial transceiver. Counters in PIT is configured in mode 0 (interrupt on terminal count) and they count the input pulses for a desired time decided by the microcontroller. The output PIT is processed by microcontroller to calculate pulse frequency. The absorbed dose of the physico-chemical medium is calculated from the pulse frequency using the pre-evaluated polynomial equation and displayed in LCD. The data is stored in an EEPROM with date and time stamp. The unit can be connected to PC via serial port to change counter configuration with new set of coefficients, set date and time and to read the saved data in memory into a notepad for further analysis. An application specific firmware is developed to introduce mathematical relation between frequency and conductivity. The change in conductivity of solution during irradiation from that of blank value (pre-irradiated solution) is further used to calculate the absorbed dose of the dosimeter. The calculated absorbed dose is displayed in the LCD. The embedded software is developed using Keil μVision4.

#### Spectrophotometer

In order to measure absorbance of ferrous–ferric solution used in Fricke dosimetry HITACHI-330 UV–Visible Spectrophotometer was used.

#### Methodology

About 2.5 mL of saturated chloroform in water was taken in the dosimeter cell. The conductance probe was vertically placed in the solution. The background reading of conductivity was recorded initially. Then the dosimeter cell was placed inside a Co-60 gamma chamber in order to irradiate the solution. Real time measurement of shift in conductivity from the blank reading, which in turn was converted to radiation dose in Gy, was displayed online in the LCD of embedded system placed outside the radiation chamber. The data can be logged simultaneously to PC whenever it is required. In some of the experiments the signal from the sensor end was allowed to transmit to the display unit which was placed about 200 m away from the radiation source.

## Results and discussion

The primary consideration behind development of sensors in our laboratory has been to generate the first electronic response in digital domain, avoiding intermediate signal processing which is normally used in conventional devices. Sensors are designed to respond very sensitively to shifts in one of the four electrical properties namely (i) ionic or electronic conductance, (ii) dielectric permeability, (iii) inductance, and (iv) electromotive force (emf) of the physical or physico-chemical systems to be probed. These four different classes of sensors, individually or in combination, offer an extensive scope for monitoring diverse parameters. Any parameter that causes change in any of the above property, directly or indirectly, becomes measurable. In all cases the sensor output is a train of rectangular pulses from which pulse frequency is computed. The pulse frequency (*f*) is a function of respective sensed parameter. Since the output of each pulsating sensor is digital pulse frequency this class of sensor is known as pulsating sensor. In the present work a pulsating conductometric sensor is deployed for online radiation dosimetry and a pulsating potentiometric sensor is used in Fricke dosimetry. The pulsating conductivity meter has certain advantages comparing to conventional conductivity meters. Some of the specific features, for which it was possible to deploy in such dedicated application, are mentioned below.

- Excellent noise immunity which is due to generation of primary signal in digital domain form,
- High resolution in measurement (less than 50 nS cm^−1^) and fast response (~20 mS),
- High precision (relative standard deviation, RSD is less than 1 %),
- High sensitivity,
- Reproducible measurement in background reading,
- Multipoint calibration approach which holds calibration for a long period,
- Signal transmission to a long distance,
- Operation with 5 V DC or even battery operated which is useful for field measurement.

**Fig. 4:**
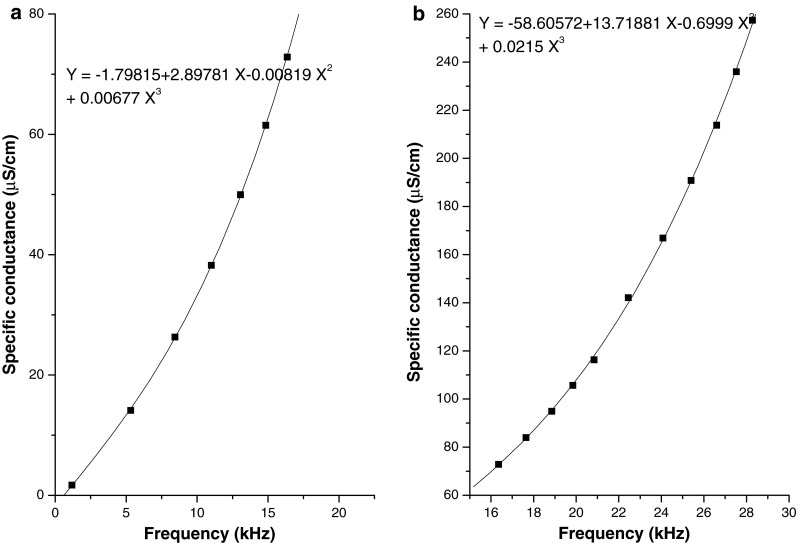
**a** Relation between pulse frequency and conductivity (range 0–80 μS cm^−1^). **b** Relation between pulse frequency and conductivity (range 80–250 μS cm^−1^)

Based on highly satisfactory performance of pulsating sensor-based conductivity monitoring instrument in many physical and physico-chemical applications [18–24] we deployed this conductivity monitoring technique to capture real time radiation induced chemical changes in a chemical dosimeter. In order to demonstrate simplicity in instrumentation for real time measurement of radiation dose saturated chloroform in water has been selected as chemical dosimeter in the present study. Our first approach for real time dosimetry relies on conductivity monitoring based on a simple, high sensitive, compact and unconventional sensing technique that operates entirely in digital domain, and developed ab initio in the laboratory. The solution conductance is directly converted to digital pulse frequency. A pair of electrodes, placed within a small volume of a dosimeter solution, senses conductivity. The digital pulses, which can be easily driven over a long distance (about 200 m) from the irradiation site, are processed for frequency information at a desired sampling rate (1 s in the present case), thus enabling time dependent measurement of a conductivity related parameter. In order to correlate frequency to conductivity for the conductivity cell deployed in the present study a multipoint calibration of conductivity probe using a series of standard KCl solutions was undertaken. Figure 4a, b shows the relation between frequency and conductivity of solution in two different ranges, <1.0–80 μS cm^−1^ and 80–250 μS cm^−1^, respectively. The performance of the conductivity meter deployed in the present study was evaluated by measuring conductivity shift with stepwise addition of 0.01 M KCl to 5 mL DW in 5 μL steps. The sensitivity, precision and resolution of measurement are found to be 370 Hz μS^−1^ cm, less than 1 % RSD and 50 nS cm^−1^, respectively. Moreover it is also observed that the calibration holds for a long duration (even up to 6 months) with periodic maintenance of electrodes.

In order to use such devices for real time dosimetry, it is necessary to establish an accurate relationship between absorbed dose and conductivity shift for a given probe. Once the radiation chemical yield (*G* value) for the product i.e. HCl, responsible for change in signal, is determined accurately, the matter is quite straight forward and information with respect to absorbed dose can be obtained directly without taking recourse to separate measurements towards calibration with respect to absorbed dose. In order to determine *G*(HCl) in the present work it is essential to know the dose rate of irradiation chamber. For precise determination of absorbed dose as a function of time, the standard approach for dose calibration through quantitative measurement of extent of gamma induced oxidation of Fe(II) to -(III) was followed. Fe(II) to -(III) conversion was measured independently by pulsating potentiometric measurement device. Ultimately using concentration of Fe^3+^ formed in each irradiated sample and taking radiation chemical yield data for Fe^3+^, *G*(Fe^3+^) as 15.5 [4, 25] the absorbed dose rate of Co-60 gamma irradiation chamber was determined using the following relation.

5 $$ {\text{Dose}}\;{\text{rate}}\,\left( {{\text{Gy}}\;{\text{h}}^{ - 1} } \right) = \left( {9.647\,\times\,10^{6} \,\times\,c} \right)/(1.024\,\times\,15.5\,\times\,t), $$

where *c* is the concentration of Fe^3+^ (moles L^−1^), 1.024 is the density of Fricke reagent (g L^−1^) [1], *t* is the time of irradiation (min).

The results are presented in Table 1. The ratio of ferric to ferrous concentration is computed from the calibration plot. The average dose rate is 391.6 ± 10.7 Gy h^−1^. The radiation dose of irradiated chamber was also determined using conventional Fricke dosimetry following spectrophotometry (refer Table 2). The radiation dose value arrived at following two independent measurement approaches agreed well.

**Table 1:** Evaluation of dose rate of Co-60 gamma chamber by Fricke dosimetry (measurement by emf approach using potentiometric measurement technique)

Irradiation time (min)	Frequencies (Hz)	Log *R*	*R* = [Fe^3+^]/[Fe^2+^]	Concentration of Fe^3+^ (μM)	Dose rate (Gy h^−1^)
10	3300	−9.4861E−01	1.1256E−01	111	405.85
20	3475	−6.2661E−01	2.3626E−01	210	383.31
30	3603	−3.9109E−01	4.0636E−01	318	386.36
40	3695	−2.2181E−01	6.0005E−01	413	376.10
50	3794	−3.9650E−02	9.1275E−01	525	382.85
60	3910	1.7379E−01	1.4921E+00	659	400.30
70	4009	3.5595E−01	2.2696E+00	764	397.80
80	4140	5.9699E−01	3.9536E+00	878	400.21

**Table 2:** Evaluation of dose rate of Co-60 gamma chamber by Fricke dosimetry (measurement by spectrophotometry)

Irradiation time (min)	Absorbance	Concentration of Fe^3+^ (μM)	Dose rate (Gy h^−1^)
20	0.632	209.0	381.08
30	0.818	311.7	378.86
40	1.03	428.7	390.83
50	1.238	543.5	396.40

**Fig. 5:**
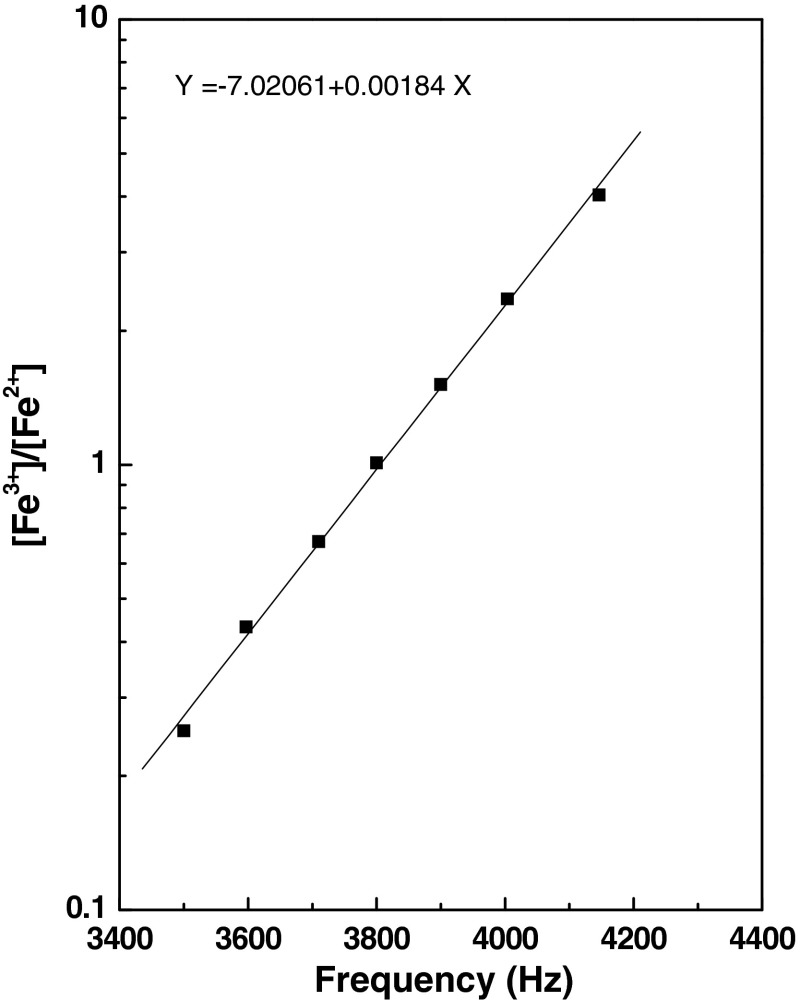
Relation between pulse frequency and ferric–ferrous ratio using digital potentiometry

In the present work an indigenously developed high resolution, fast response potentiometric measurement technique was used to measure ferric concentrations besides spectrophotometry in order to determine the dose rate of irradiation chamber. The specific features of potential monitoring instrument have been mentioned elsewhere [26, 27]. The instrument is designed in such a way that the emf generated at the interface of electrode and electrolyte is directly converted to digital pulse frequency using a specially designed voltage to frequency (*V*–*f*) converter. Hence the emf is directly proportional to pulse frequency. Based on the potential measurement of a series of standard Fe^2+^–Fe^3+^ solutions of various ratios (prepared in a mixture of 0.4 M H_2_SO_4_ and 1 mM NaCl solutions’ matrix as per the composition of Fricke dosimeter) a calibration plot was prepared following Nernst equation. Figure 5 shows the relation between frequency and ferric–ferrous ratio ([Fe^3+^]/[Fe^2+^]). This relation was used to evaluate the concentrations of Fe^3+^ formed during irradiation as given in Table 1.

In order to evaluate radiation chemical yield value of HCl (*G*(HCl)) a series of saturated CHCl_3_ solutions were irradiated for specific durations. The concentration of trace HCl formed during irradiation of each sample was determined following two independent approaches. In the first approach the conductivity shift of each irradiated solution was measured by applying temperature normalization at 25 °C. In order to do so an in-house built mini capacitance-based temperature sensor was placed in solution and it was connected to a separate channel. The temperature measured online was used in the system software to compute conductivity at 25 °C. The concentration of HCl was computed using the following relation.

6 $$ c = (\kappa /(1,000\,\times\,\lambda )), $$

where *c* is the concentration (g equiv L^−1^), *κ* is the specific conductance (μS cm^−1^), *λ* is the equivalent conductance (S cm^2 ^g equiv^−1^)

In this work since concentration of HCl is extremely low *λ* is taken as 426 S cm^2^ g equiv^−1^ i.e. the same as equivalent conductance of HCl at infinite dilution (sum of equivalent conductance of H^+^ and Cl^−^). In another independent measurement campaign the concentration of HCl of each irradiated sample was determined following titrimetric approach using pulsating conductometric titration technique developed in-house. *G*(HCl) in each case was calculated using the following relation.

7 $$ G({\text{HCl}}) = \left( {9.647\,\times\,10^{6} \,\times\,[{\text{HCl}}]} \right)/D, $$

where [HCl] is the concentration of HCl in moles L^−1^, *D* is the absorbed dose in Gy.

Tables 3 and 4 summarize the results for determination of *G* value of HCl using two independent techniques i.e. conductivity measurement approach and conductometric titration technique, respectively. Based on these results the *G*(HCl) for chloroform dosimetry is evaluated as 10.2 in the present study. The relation between absorbed dose and shift in conductivity is derived by using following computational approach.

**Table 3:** Determination of *G* value of HCl in saturated chloroform dosimeter using conductivity measurement approach

Irradiation time (s)	Sp. cond. before irradiation (μS cm^−1^)	Sp. cond. after irradiation (μS cm^−1^)	Sp. cond. due to HCl (μS cm^−1^)	Concentration of HCl (μM)	Absorbed dose (Gy)	*G*(HCl)
300	1.2199E+00	1.5184E+01	1.3964E+01	32.8	32.433	9.75
600	1.2199E+00	3.0874E+01	2.9654E+01	69.6	64.867	10.35
900	1.2199E+00	4.5777E+01	4.4557E+01	104.6	97.30	10.37
900	1.2428E+00	4.5665E+01	4.4422E+01	104.3	97.30	10.34
300	1.2326E+00	1.5805E+01	1.4573E+01	34.2	32.433	10.18
600	1.2071E+00	2.9680E+01	2.8473E+01	66.8	64.867	9.94
1,200	1.2071E+00	5.9949E+01	5.8742E+01	137.9	129.73	10.25
1,800	8.5389E−01	8.8970E+01	8.8116E+01	206.9	194.60	10.25
3,600	8.6172E−01	1.7920E+02	1.7834E+02	418.6	389.20	10.38
1,800	8.5128E−01	8.8166E+01	8.7315E+01	205	194.60	10.16
2,400	8.5128E−01	1.1703E+02	1.1618E+02	272.7	259.47	10.14
3,000	8.5128E−01	1.4617E+02	1.4531E+02	341.1	324.33	10.15
3,600	8.5128E−01	1.7284E+02	1.7199E+02	403.7	389.20	10.01

**Table 4:** Determination of *G* value of HCl in saturated chloroform dosimeter using conductometric titration technique

Irradiation time (min)	Absorbed dose (Gy)	End point	Drop volume (mL)	NaOH required (mL)	Concentration of NaOH (mM)	Concentration of HCl (mM)	*G*(HCl)
15	97.3	42	0.007	0.294	0.87	0.102	10.14
30	194.6	45	0.007	0.315	1.74	0.219	10.86
60	389.2	35	0.007	0.245	0.435	0.0426	10.57
20	129.73	26	0.013	0.338	1.0	0.135	10.05
10	64.87	13	0.013	0.169	1.0	0.0067	10.05

The concentration of HCl, formed due to induced radiation effect is expressed as

8 $$ [{\text{HCl}}] = \Updelta \kappa /(426\,\times\,1,000), $$

where [HCl] is the concentration of HCl in moles L^−1^, Δ*κ* is the shift in conductivity in μS cm^−1^.

9 $$ {\text{Dose}}\,({\text{in}}\;{\text{Gy}}) = \left\{ {\left( {[{\text{HCl}}]\,\times\,9.647\,\times\,10^{8} } \right)/10.2} \right\}\,\times\,0.01. $$

Substituting [HCl] in Eq. 9 the dose is expressed as

10 $$ {\text{Dose}}\;({\text{in}}\;{\text{Gy}}) = \Updelta \kappa \,\times\,2.22. $$

This relation between shift in conductivity and radiation dose was used in the embedded processor to get direct display of radiation dose which would be useful for field measurement. As it is possible to determine *G*(HCl) for saturated chloroform dosimetry, this value can be used to determine the dose rate of Co-60 gamma irradiation chamber without depending upon any external technique.

The standalone embedded instrument deployed in the present work does the following functions:

- Background conductivity measurement

It measures background conductivity value of the chemical dosimeter and calculates average of 10 values recorded at regular interval and stores it in memory.

(b) Real time measurement of conductivity shift

It continuously measures the conductivity shift of the solution due to irradiation in radiation field. It measures actual conductivity of the solution at that time and deducts background value from the actual conductivity. It stores Δ*κ* value.

$$ \Updelta \kappa = \kappa_{\text{actual}} - \kappa_{\text{background}} . $$

(c) Measurement of radiation dose from conductivity

It converts Δ*κ* into radiation in dose by using suitable multiplication factor.

**Fig. 6:**
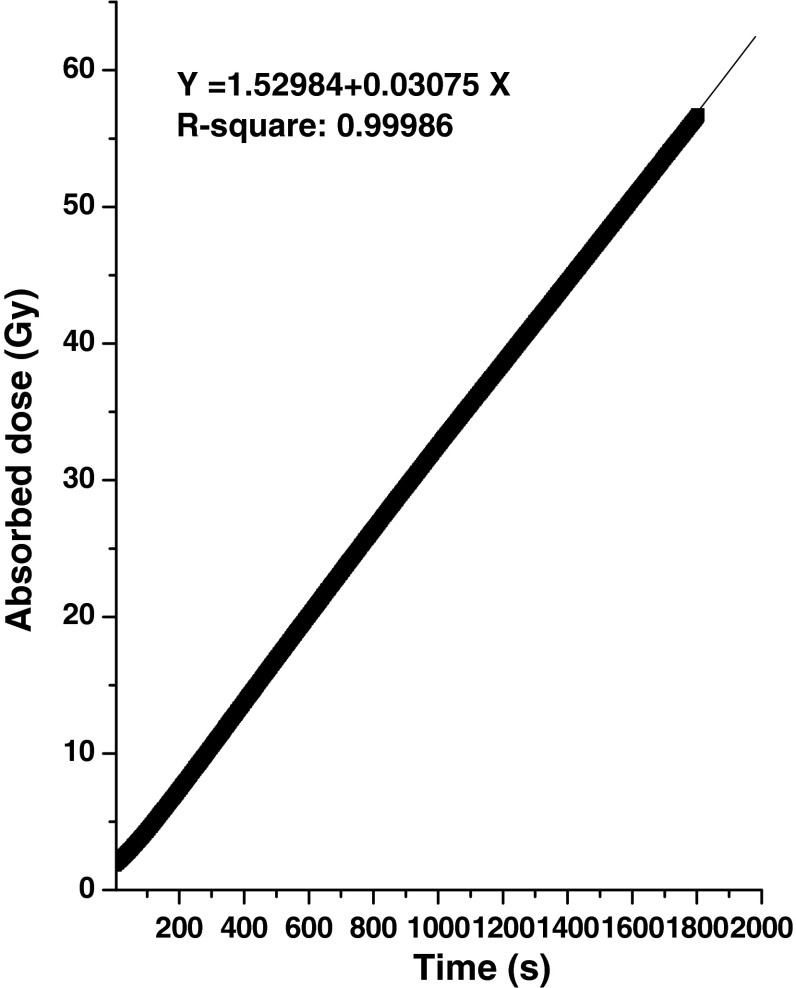
Linear increase in radiation dose with respect to irradiation time in a typical online measurement campaign

Before deployment of the instrument in real time measurement of radiation dose some offline measurements were also conducted by irradiating a series of saturated chloroform solutions for fixed durations. In each case the conductivity shift from pre-irradiated solution was measured and the absorbed dose was computed using *G*(HCl) as 10.2. The results were presented in Table 5. These results reveal that radiation dose with respect to time of irradiation show a linear trend with regression coefficient (*R* ^2^) 0.99983. The present technique could be successfully deployed for measurement of absorbed dose when the dose level remains within 400 Gy. This specially designed embedded instrument was further used for real time measurement of radiation dose obtained from Co-60 irradiation chamber in several measurement campaigns. Results from a typical online measurement campaign are illustrated in Fig. 6. The data shows linear increase in radiation dose with respect to irradiation time (*R* ^2^ 0.99986).

**Table 5:** Summary of results from a series of irradiated saturated chloroform solutions (irradiated in Co-60 gamma chamber)

Time of irradiation (min)	Conductivity of pre-irradiated solution (μS cm^−1^)	Conductivity after irradiation (μS cm^−1^)	Shift in conductivity (μS cm^−1^)	Concentration of HCl (μM)	Dose (Gy)
1.5	1.22	5.73	4.51	10.6	10.01
8	1.13	24.03	22.9	53.8	50.84
12	1.18	35.23	34.05	79.9	75.6
24	1.25	71.6	70.35	165.0	156.19
36	1.23	103.46	102.23	240.0	226.97
52	1.16	151.68	150.52	353.0	334.18
64	1.2	186.2	185.0	434.0	410.73

## Conclusion

In the present work real time measurement of absorbed dose from gamma irradiation field using a chemical dosimeter with simple instrumentation approach has been demonstrated. A high resolution fast response conductivity monitoring device was deployed to capture minor shift in conductivity due to radiation induced chemical change in the saturated chloroform solution used as chemical dosimeter. A specially designed embedded processor was used to capture signal directly from the sensor and converted it to the practical dose unit. Many real time measurement campaigns were successfully conducted in order to build up the confidence for deployment of the device in field measurement. Besides realisation of real time dosimeters, the work also led to a simpler and more convenient user friendly tool for dose calibration compared to conventional procedure such as by Fricke dosimetry. The campaign has also proved usefulness of our digital electrochemical devices for radiation chemistry work in general. The present work has been taken up in order to realize a conductivity-based real time dosimeter with simple instrumentation. With the present design we have measured radiation dose up to 400 Gy. However there is scope of pursuing this research to extend the technique for high dose measurement by modifying the instrumentation. Moreover the effect of dose rate on saturated chloroform dosimeter is being investigated.
